# Phytotelm and anthropogenic mosquitoes: community structure in natural and artificial habitats in an Atlantic Forest agroforestry system, Rio de Janeiro, Brazil

**DOI:** 10.1007/s00114-026-02162-8

**Published:** 2026-09-16

**Authors:** Cecília Ferreira de Mello, Gerson Azulim Müller, Wellington Thadeu de Alcantara Azevedo, Hélcio Reinaldo Gil-Santana, Thamires Rezende Araújo, Roger Pimentel, Jeronimo Alencar

**Affiliations:** 1 Fundação Oswaldo Cruz, Fiocruz, Instituto Oswaldo Cruz, Laboratório de Diptera, Avenida Brasil 4365, Manguinhos, Rio de Janeiro, RJ CEP: 21040-360 Brasil; 2 Instituto Federal de Educação, Ciência e Tecnologia Farroupilha, Campus Panambi, Rua Erechim 860, Planalto, Panambi, RS CEP 98280-000 Brasil; 3 Departamento de Microbiologia e Parasitologia, Instituto Biomédico; Centro de Ciências Biológicas e da Saúde; Universidade Federal do Estado do Rio de Janeiro, Avenida Pasteur, 458, Urca, Rio de Janeiro, CEP: 22290-240 Brasil

**Keywords:** Culicidae, Diptera, Ecotone, Phytotelmata, Larvae

## Abstract

Agroforestry systems (AFSs) combine agricultural crops with native or exotic trees to promote sustainable production while creating heterogeneous landscapes with diverse fauna microhabitats. In the Atlantic Forest biome, how these systems shape immature mosquito (Culicidae) communities and vector dynamics remains poorly understood. We characterized the immature mosquito community (larvae and pupae) across five larval habitat types, three natural (bromeliads, bamboo internodes, and peach palm leaf bracts) and two artificial (a plastic tarp and a water barrel), in an AFS in the state of Rio de Janeiro, Brazil. A total of 5,314 specimens belonging to 38 species distributed in 10 genera were collected. Natural larval habitats, particularly bromeliads and bamboo internodes, presented the highest species richness and harbored most of the exclusive taxa. Artificial habitats harbored fewer species, among them *Culex quinquefasciatus*, a synanthropic species of public health importance. Other epidemiologically relevant mosquitoes were *Haemagogus leucocelaenus*, *Haemagogus janthinomys*, and *Aedes albopictus*, the last of these a highly generalist species recorded in both natural and artificial larval habitats. Our results show that AFSs act as interface zones between forest and human-altered environments, with significant epidemiological implications: such systems can favor arbovirus spillover and bring vectors, human populations, and wildlife reservoirs into closer contact.

**Supplementary Information:**

The online version contains supplementary material available at https://doi.org/10.1007/s00114-026-02162-8.

## Introduction

The family Culicidae comprises mosquitoes, the insects of greatest medical and veterinary importance, owing to their hematophagous habits, wide and persistent distribution, and role as vectors of pathogens responsible for diseases such as dengue, malaria, and yellow fever, thereby posing major challenges to public health (Calderón-Peláez et al. 2023). In Brazil, the emergence and reemergence of diseases such as dengue, Zika, chikungunya, and yellow fever have highlighted the pressing need to understand the ecological factors that determine the distribution and abundance of vector species (Daudt-Lemos et al. 2025). Among these factors, the availability and type of larval habitats are central elements, since the immature stages of Culicidae are aquatic and strongly dependent on the physicochemical characteristics, permanence, and origin of water bodies (Martínez-Barciela et al. 2025).

Mosquito larval habitats are traditionally classified as natural (such as bromeliads, bamboo internodes, tree holes, and temporary pools) or artificial (tires, cans, water tanks, plastic tarps, and disposable containers) (Forattini 2002). While natural larval habitats sustain a fauna that is frequently specialized and highly endemic, artificial habitats tend to select for generalist and synanthropic species, many of them efficient vectors of human pathogens (Alencar et al. 2016). The Brazilian Atlantic Forest, a severely fragmented biodiversity hotspot, presents a particularly dynamic interface between forest remnants and human-modified areas with respect to culicid communities (Barbosa et al. 2025).

Over the past few decades, agroforestry systems (AFSs) have been promoted as sustainable alternatives to deforestation and monoculture, reconciling agricultural production with biodiversity conservation (Zha and Zhang 2025). These systems combine native or exotic tree species with agricultural crops, creating heterogeneous vegetation mosaics and, consequently, a diversity of microhabitats for fauna, including mosquitoes (Silva et al. 2026). For instance, species of *Sabethes*, *Limatus*, and *Psorophora* have been recorded in a cocoa AFS in Bahia, showing that these landscapes can host medically relevant mosquito taxa (Catenacci et al. 2017). However, little is known about how agroforests influence the community structure of immature Culicidae, particularly in comparison with continuous forest environments or open areas. The presence of plants such as açaí (*Euterpe oleracea* Mart.) and peach palm (*Bactris gasipaes* Kunth), whose leaves form phytotelmata, combined with artificial materials used in agricultural management (plastic tarps and water barrels), may create a complex larval habitat matrix with varying degrees of attractiveness for different species.

Despite advances in knowledge of culicids in natural phytotelmata of the Atlantic Forest, significant gaps remain: most studies have focused on only one or two larval habitat types, hindering direct comparisons; AFSs remain undersampled in entomological surveys; and analyses integrating species richness, abundance, and habitat specialization across natural and artificial larval habitats within the same landscape mosaic are lacking (Camara et al. 2020). This gap is particularly critical because agroforests are expanding as a policy for ecological restoration and income generation in the Atlantic Forest, becoming potential environments for contact between sylvatic vectors and humans (Silva et al. 2026).

The present study aimed to characterize the immature mosquito community (larvae and pupae) in an AFS in the municipality of Silva Jardim, state of Rio de Janeiro, within the Atlantic Forest domain, across five larval habitat types: three natural (bromeliads, bamboo internodes, and peach palm leaf bracts) and two artificial (a plastic tarp and a water barrel). Specifically, we sought to: describe species richness, abundance, and composition in each habitat type; test whether species distribution across larval habitats is random or indicative of habitat specialization; and compare faunal similarity between natural and artificial larval habitats. The central hypothesis is that natural larval habitats sustain greater richness and a higher number of exclusive species, whereas artificial larval habitats harbor lower richness but are dominated by generalist vector species, such as *Ae. albopictus*. The results may contribute to understanding mosquito ecology in agroecological landscapes and provide a basis for more targeted vector surveillance and control strategies.

## Materials and methods

### Study area

**Fig. 1:**
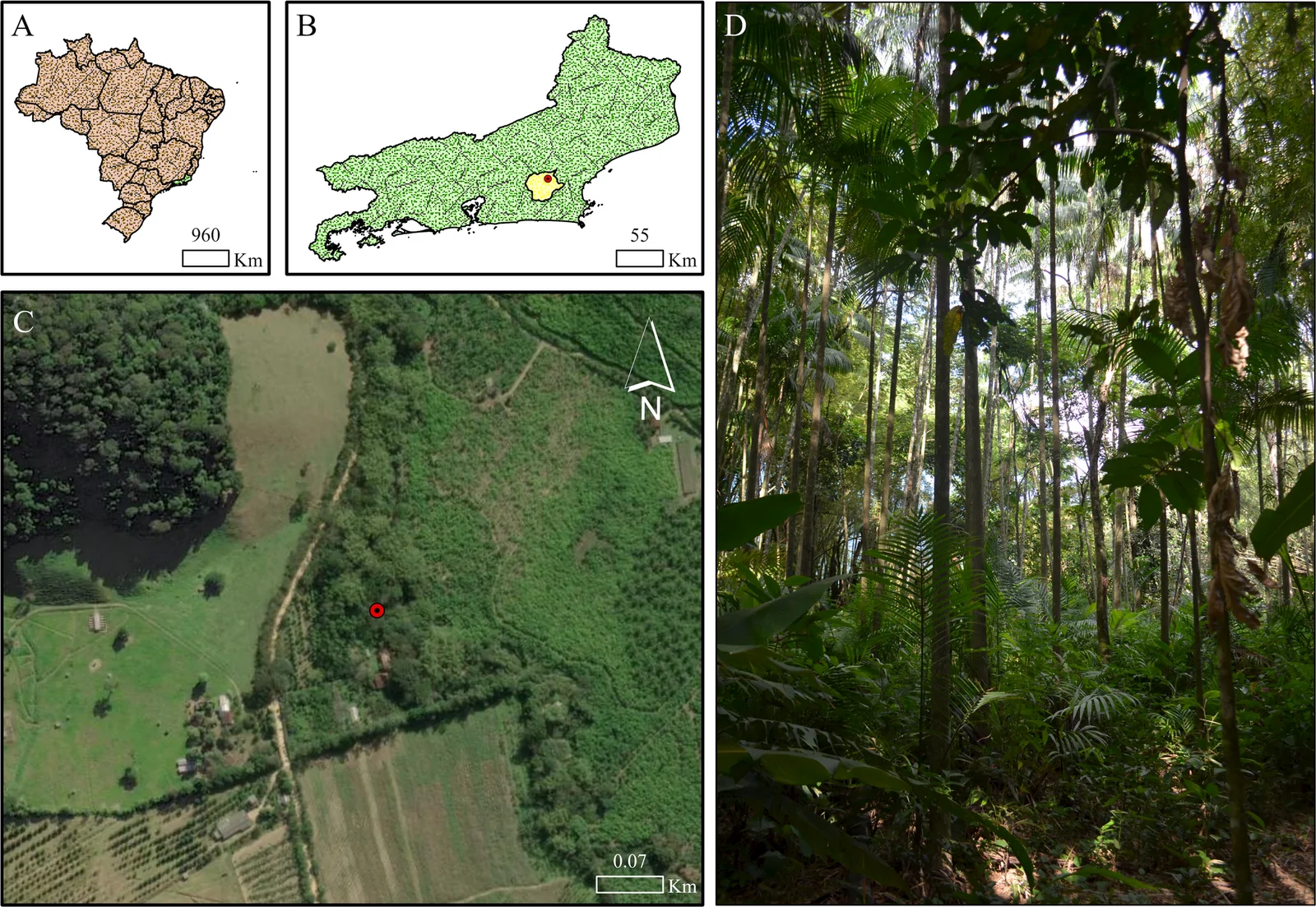
Location and characterization of the study area at Terra Boa Farm, municipality of Silva Jardim, state of Rio de Janeiro, Brazil. Panoramic view of the wetland area at the study site. Map created using ArcGis Pro 3.0.1 (ESRI, Redlands, CA, USA) SIRGAS 2000

Sampling conducted at Terra Boa Farm (22°29’42.7"S, 42°21’41.8"W), Silva Jardim, Rio de Janeiro, Brazil, in the Atlantic Forest phytogeographic domain (Fig. 1). Spatial data processing, mapping, and area calculations were performed in ArcGIS Pro 3.0.1 (ESRI, Redlands, CA, USA), using the SIRGAS 2000 datum and UTM zone 23 S. The study area consisted of an agroforestry plot of approximately 3.08 ha and a perimeter of 825 m. This is an agroecological system combining the cultivation of agricultural species with native trees, promoting biodiversity, soil recovery, and environmental sustainability (Gliessman 2000; Altieri 2012). The area comprised an intercropped plantation of açaí (*E. oleracea*) and peach palm (*B. gasipaes*), species typical of AFSs in the region, which, in addition to generating income, contribute to the maintenance of local fauna and flora (Young 1997). This environment was selected given the growing importance of agroforests as modified habitats that may harbor arbovirus vectors, warranting entomological surveillance studies.

### Collection and rearing of immature stages

Collections were carried out monthly from June 2022 to December 2025, over 43 sampling events totaling 86 h of sampling effort. Culicid immatures (larvae and pupae) were collected using suction bottles fitted with two tubes, following a technique adapted from Müller et al. (2014), which improved sampling precision. The aquatic contents of each larval habitat were poured into white trays, and larvae and pupae were transferred using plastic Pasteur pipettes. We sampled natural habitats (bamboo internodes, bromeliads, and peach palm leaf bracts) as well as artificial habitats already present in the area: a 200-L polyethylene water barrel and a polyethylene tarp laid flat on the ground beside the bromeliads, where depressions in the tarp accumulated rainwater (Supplementary Material 1: Fig. S1).

At each visit we examined five bromeliads, five bamboo internodes, one peach palm leaf bract, one plastic tarp, and one water barrel. None of these habitats, natural or artificial, were introduced or deliberately placed by the research team. Natural habitats were emptied completely, removing 100% of their water volume. In bamboo internodes previously cut within the study area, water was collected with siphon bottles or by sectioning the internodes directly (Müller et al. 2014). Artificial habitats were not subject to a fixed volume limit; they were emptied exhaustively so that every immature mosquito present was collected.

Collected larvae and pupae were placed in 250-mL plastic bags (Whirl-Pak^®^, BioQuip^®^), labeled with information on locality, date, larval habitat type, and water volume, and transported to the laboratory. In the laboratory, immatures were maintained in plastic containers filled with water from the original larval habitat. During the rearing period, water was replaced with dechlorinated water and larvae were fed fish food (Tetramin^®^), following the protocol of Gerberg et al. (1994). Adult emergence was monitored daily until complete pupation and eclosion.

### Specimen processing and identification

Taxonomic identification was carried out by direct observation of external morphological characters under a light microscope (Leica DMD108^®^), using the dichotomous keys of Lane (1953), Consoli and Oliveira (1994), and Forattini (2002), and Oliveira-Christe and Marrelli (2025) for the subgenus *Cx.* (*Microculex*). To ensure accurate species-level identification, particularly within *Cx.* (*Microculex*), males from the sampled larval habitats were dissected and identified based on their genital morphology. Generic and subgeneric name abbreviations follow (Reinert 2009). Voucher specimens will be deposited in the Entomological Collection of the Instituto Oswaldo Cruz (Fiocruz), Rio de Janeiro, Brazil.

### Data analysis

Individual-based species accumulation curves (interpolation and extrapolation) with 95% confidence intervals, computed using the iNEXT package in R (Hsieh et al. 2016). Curves were extrapolated to 8,350 individuals for the pooled natural larval habitats and to 4,062 for bromeliads, the largest single habitat sampled; a Venn diagram to visualize species overlap between natural and artificial larval habitats; bipartite networks (species-by-larval-habitat interactions) to identify specialization patterns; and the Jaccard dissimilarity index with cluster analysis (UPGMA) to assess similarity in species composition among larval habitat types. All analyses were performed in the R statistical environment (R Core Team 2025) at a significance level of *p* < 0.05.

## Results

A total of 5,314 specimens belonging to 38 species and 10 genera were collected. The most abundant genus was *Culex*, accounting for 53.8% of all collected specimens, followed by *Limatus* (16.6%) and *Wyeomyia* (16.2%). Five species, *Cx. iridescens*, *Cx. pleuristriatus*, *Li. durhamii*, *Wy. edwardsi*, and *Ae. albopictus*, accounted for 79.8% of all captured specimens (Table 1).

**Table 1:** Mosquito taxa collected from June 2022 to December 2025 in larval habitats located at Terra Boa Farm, Silva Jardim, Rio de Janeiro, Brazil

Larval habitat:	Natural						Artificial			
	Bromeliad (*n* = 39)		Bamboo (*n* = 37)		Palm (*n* = 7)		Plastic canvas (*n* = 20)		Water tank (*n* = 4)	
Species	n	%	n	%	n	%	n	%	n	%
*Aedeomyia* (*Aedeomyia*) *squamipennis* (Lynch Arribálzaga, 1878)	-	-	-	-	2	0.40	-	-	-	-
*Aedes* (*Stegomyia*) *albopictus* (Skuse, 1894)	29	1.43	46	2.80	10	2.01	393	35.18	14	63.64
*Aedes* (*Ochlerotatus*) *scapularis* (Rondani, 1848)	-	-	1	0.06	-	-	-	-	-	-
*Aedes* (*Protomacleaya*) *terrens* (Walker, 1856)	2	0.10	66	4.01	-	-	-	-	-	-
*Culex ocellatus* Theobald, 1903	1	0.05	-	-	-	-	-	-	-	-
*Culex* (*Carrollia*) *iridescens* (Lutz, 1905)	14	0.69	1.192	72.42	24	4.82	-	-	-	-
*Culex* (*Culex*) *mollis* (Dyar & Knab, 1906)	3	15	6	0.37	155	31.13	71	6.36	-	
*Culex* (*Culex*) *quinquefasciatus* (Say, 1823)	-	-	-	-	-	-	70	6.27	-	-
*Culex* (*Culex*) sp.	-	-	-	-	10	2.01	-	-	-	-
*Culex* (*Melanoconion*) sp.	-	-	-	-	-	-	3	0.27	2	9.10
*Culex* (*Microculex*) *inimitabilis* (Dyar & Knab, 1906)	2	0.10	-	-	-	-	-	-	-	-
*Culex* (*Microculex*) *neglectus* (Lutz, 1904)	-	-	32	194	-	-	-	-	-	-
*Culex* (*Microculex*) *pleuristriatus* (Theobald, 1903)	1.029	50.67	82	4.98	-	-	-	-	-	-
*Culex* (*Microculex*) *retrosus* (Lane & Whitman, 1951)	129	6.35	1	0.06	-	-	-	-	-	-
*Culex* (*Microculex*) *worontzowi* (Pessoa & Galvão, 1936)	21	1.03	-	-	-	-	-	-	-	-
*Culex* spp.	8	0.39	3	0.18	-	-	-	-	1	4.55
*Haemagogus* (*Haemagogus*) *janthinomys* (Dyar, 1921)	-	-	1	0.06	-	-	-	-	-	-
*Haemagogus* (*Conopostegus*) *leucocelaenus* (Dyar & Shannon, 1924)	-	-	18	1.09	-	-	-	-	-	-
*Limatus durhamii* (Theobald, 1901)	4	0.20	4	0.24	204	40.96	542	48.52	3	13.64
*Limatus flavisetosus* (Oliveira Castro, 1935)	-	-	-	-	-	-	28	2.51	-	-
*Limatus pseudomethisticus* (Bonne-Wepster & Bonne, 1920)	-	-	-	-	91	18.27	7	0.63	-	-
*Lutzia* (*Lutzia*) *bigoti* (Bellardi, 1862)	-	-	-	-	-	-	-	-	1	4.55
*Sabethes* (*Peytonulus*) *identicus* (Dyar & Knab, 1907)	-	-	19	1.15	-	-	-	-	-	-
*Toxorhynchites* (*Lynchiella*) *bambusicola* (Lutz & Neiva, 1913)	-	-	1	0.06	-	-	-	-	-	-
*Toxorhynchites* sp.	3	0.15	5	0.30	-	-	-	-	-	-
*Toxorhynchites* (*Lynchiella*) *pusillus* (Costa Lima, 1931)	-	-	2	0.12	-	-	-	-	-	-
*Toxorhynchites* (*Ankylorhynchus*) *trichopygus* (Wiedemann, 1828)	11	0.54	-	-	-	-	-	-	-	-
*Toxorhynchites* (*Megarhina*) *violaceus* (Wiedemann, 1820)	2	0.10	-	-	-	-	-	-	-	-
*Trichoprosopon compressum* (Lutz, 1905)	-	-	30	1.82	-	-	-	-	-	-
*Trichoprosopon digitatum* (Rondani, 1948)	-	-	52	3.16	-	-	-	-	-	-
*Trichoprosopon* sp.	3	0.15	-	-	-	-	-	-	-	-
*Wyeomyia* (*Phoniomyia*) *edwardsi* (Lane & Cerqueira, 1942)	630	31.02	20	1.22	-	-	2	0.18	-	-
*Wyeomyia* (*Wyeomyia*) *arthrostigma* (Lutz, 1905)	14	0.69	56	3.40	1	0.20	-	-	-	-
*Wyeomyia* (*Spilonympha*) *bourrouli* (Lutz, 1905)	39	1.92	-	-	-	-	-	-	-	-
*Wyeomyia* (*Cruzmyia*) *dyari* (Lane & Cerqueira, 1942)	1	0.05	-	-	-	-	-	-	-	-
*Wyeomyia* (*Wyeomyia*) *medioalbipes* Lutz, 1904	65	3.20	-	-	-	-	-	-	1	4.55
*Wyeomyia* (*Miamyia*) *oblita* (Lutz, 1904)	7	0.35	-	-	-	-	-	-	-	-
*Wyeomyia* sp.	14	0.69	9	0.55	1	0.2	1	0.09	-	-
Total	2.031	100	1.646	100	498	100	1.117	100	22	100

**Fig. 2:**
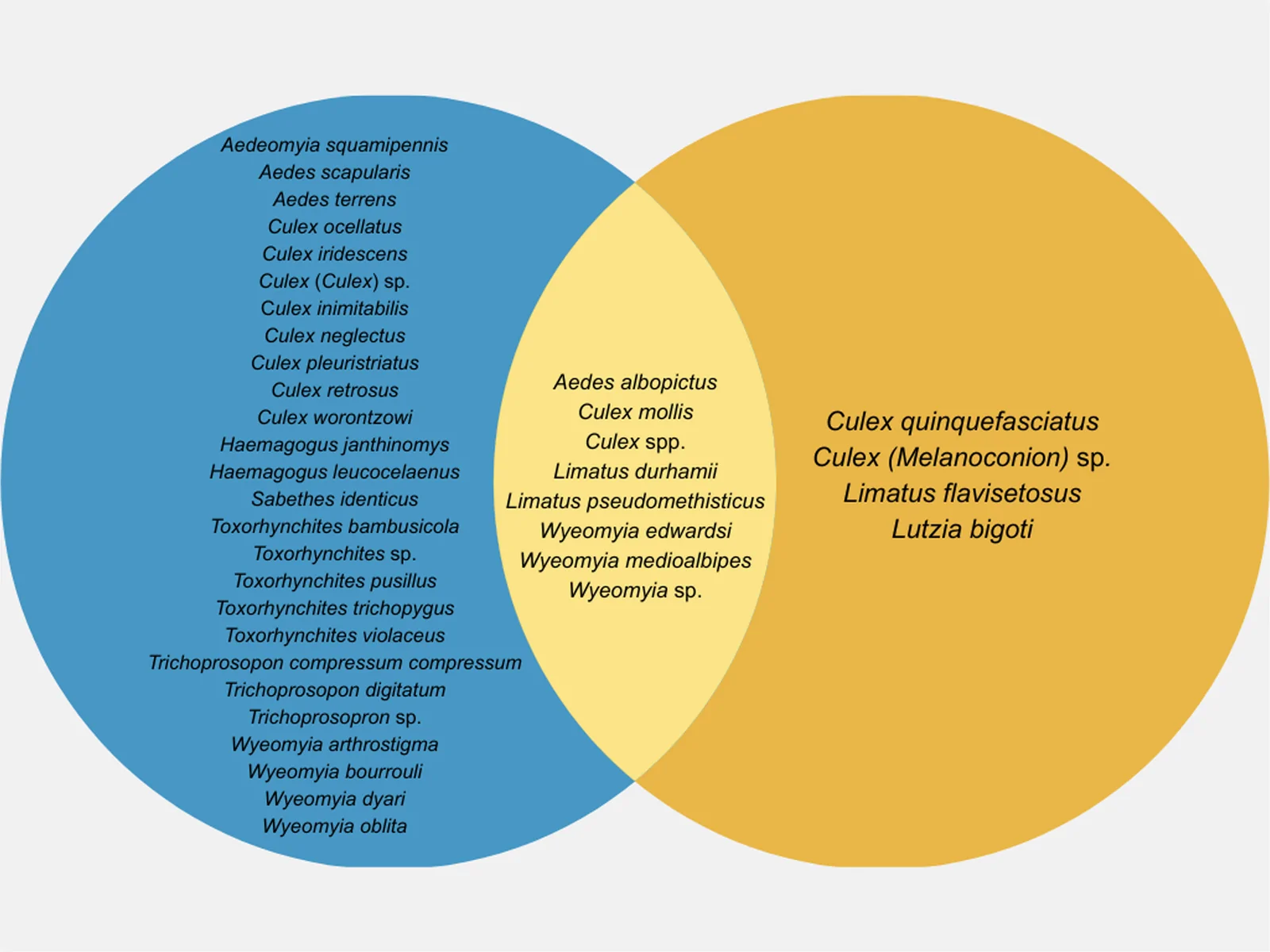
Venn diagram of species distribution across larval habitat groups, showing the number of mosquito species exclusive to natural larval habitats (blue), exclusive to artificial larval habitats (orange), and shared between both groups (yellow)

**Fig. 3:**
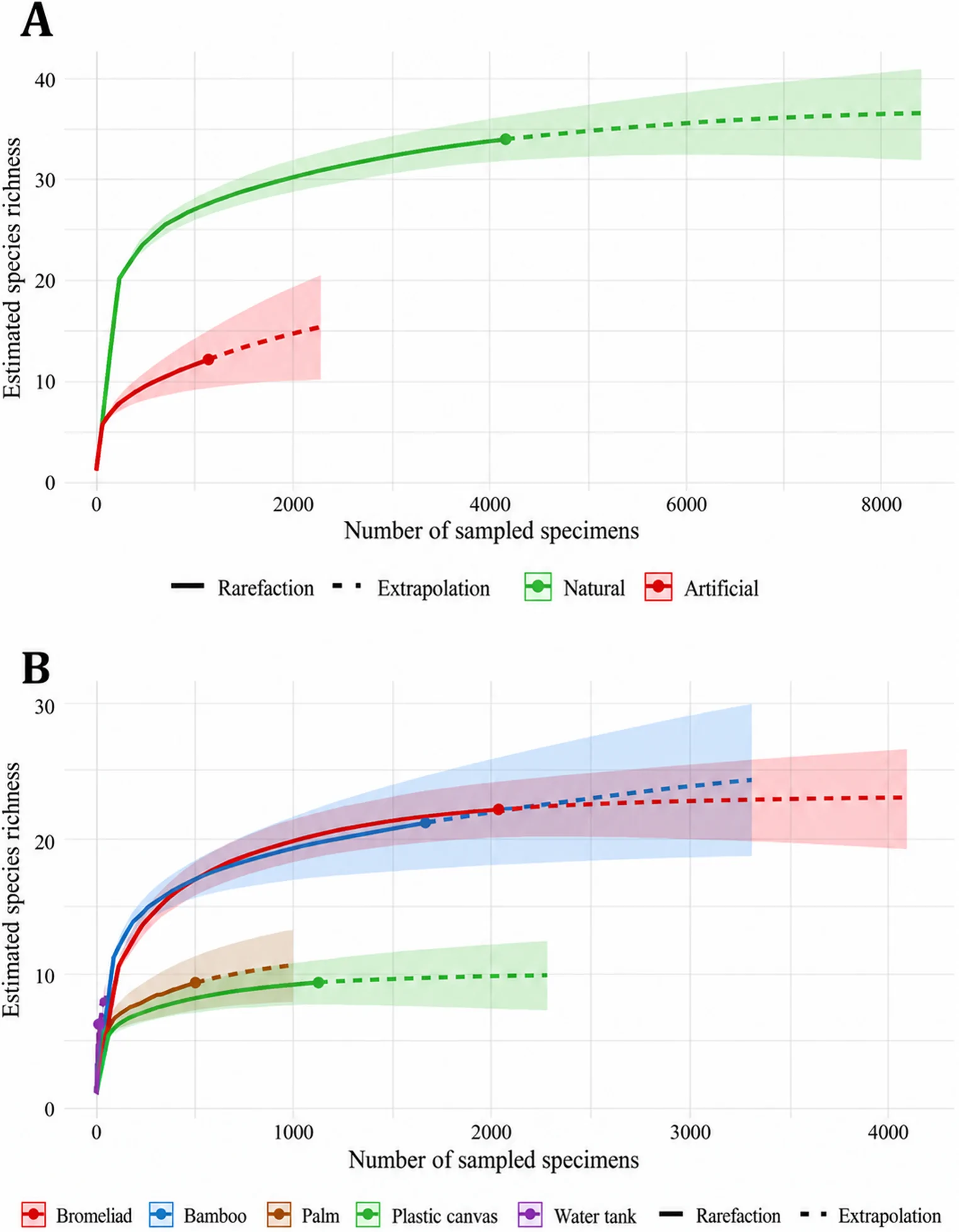
Individual-based species accumulation curves for mosquito species collected from larval habitats at Terra Boa Farm, Silva Jardim, Rio de Janeiro, Brazil. (**A**) Natural and artificial larval habitat groups. (**B**) Five larval habitat types. Solid lines, circles, and dashed lines represent interpolated, observed, and extrapolated species richness, respectively. Shaded areas indicate 95% confidence intervals

Larval habitats classified as natural (bromeliads, bamboo internodes, and peach palms) yielded the greatest abundance of immature mosquitoes, with 4,175 specimens (78.6%). Artificial larval habitats (plastic tarp and water barrel) accounted for 1,139 specimens (21.4%). In the natural habitat group, three species stood out as the most abundant: *Cx. iridescens* (1,230; 29.5%), *Cx. pleuristriatus* (1,111; 26.6%), and *Wy. edwardsi* (650; 15.6%). In the artificial group, two species predominated: *Li. durhamii* (545; 47.8%) and *Ae. albopictus* (407; 35.7%). Twenty-six species occurred exclusively in natural larval habitats, totaling 2,873 specimens (54.0%). Four species were restricted to artificial larval habitats (104; 2.0%). Eight species were collected in both habitat groups, totaling 2,337 specimens (44.0%) (Fig. 2). Individual-based species accumulation curves for both habitat groups indicated that natural larval habitats supported significantly greater species richness than artificial ones (Fig. 3A).

**Fig. 4:**
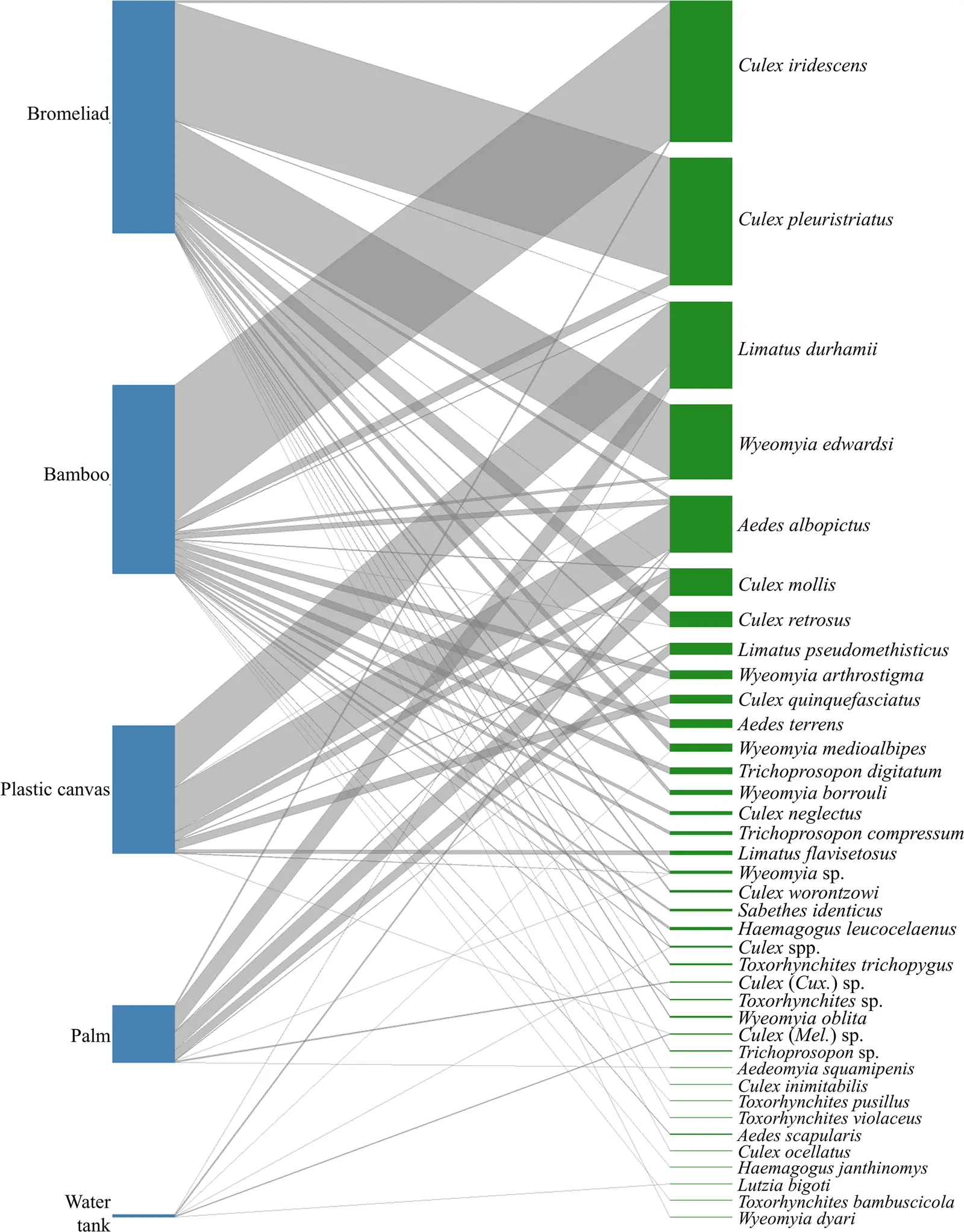
Bipartite interaction network between mosquito species and larval habitat types. Each line connects a mosquito species to a larval habitat type in which it was recorded; line and bar width are proportional to the number of individuals collected

When larval habitats were analyzed separately, mosquitoes were collected predominantly from bromeliads, totaling 2,031 individuals (38.2%) belonging to 22 species. In bamboo internodes, 1,646 individuals (31.0%) belonging to 21 species were collected, while 1,117 specimens (21.0%) belonging to nine species were collected from the plastic tarp. Peach palm larval habitats yielded 498 specimens (9.4%) belonging to nine species. Only 22 individuals (0.4%) of six species were collected from the water barrel (Table 1). The species-by-larval-habitat bipartite networks indicated that the mosquito community in bromeliads was composed predominantly of *Cx. pleuristriatus* and *Wy. edwardsi*. In bamboo internodes, *Cx. iridescens* was dominant; in the plastic tarp, *Li. durhamii* and *Ae. albopictus* predominated. In peach palm larval habitats, three species were most numerous: *Li. durhamii*, *Li. pseudomethisticus*, and *Cx. mollis*. In the water barrel, despite the low overall abundance, *Ae. albopictus* and *Culex (Mel.)* sp. were the most frequently recorded species (Fig. 4; Supplementary Table S2).

Species accumulation curves for each larval habitat type indicated that bromeliads and bamboo internodes supported greater species richness than peach palms and the plastic tarp. For the water barrel, extrapolation was not possible due to the low number of specimens collected (Fig. 3B).

**Fig. 5:**
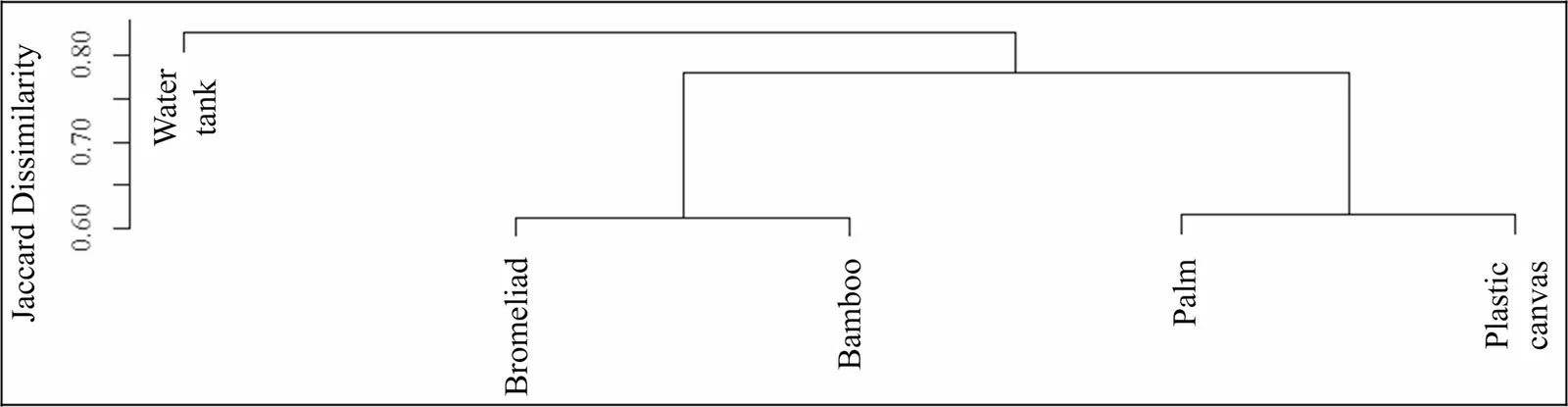
Cluster analysis (UPGMA) based on the Jaccard dissimilarity index of mosquito species composition across five larval habitat types at Terra Boa Farm, Silva Jardim, Rio de Janeiro, Brazil. Scale ranges from 0 (identical composition) to 1 (no shared species)

Cluster analysis based on the Jaccard dissimilarity index revealed greater similarity in species composition between bromeliads and bamboo internodes (0.613) and between peach palm and the plastic tarp (0.615). The water barrel formed a cluster clearly distant from the other four larval habitat types (bromeliad, 0.833; bamboo, 0.875; peach palm, 0.846; plastic tarp, 0.750; Fig. 5).

## Discussion

Our results supported the hypothesis that natural larval habitats in the Terra Boa Farm AFS harbored significantly higher species richness and a greater number of exclusive species than artificial ones (34 vs. 12 species; 26 vs. 4 exclusive taxa). The 38 species recorded across 10 genera indicate that this AFS maintains a complex, species-rich mosquito community comparable to, and in some cases exceeding, that observed in preserved Atlantic Forest remnants in southeastern and southern Brazil (Marchi et al. 2010; Silva et al. 2024).

This high diversity aligns with evidence from national and international studies showing that agroforestry and other tree-covered agricultural systems can sustain diverse mosquito communities (Abella-Medrano et al. 2015; Catenacci et al. 2018). By combining agricultural crops with native or exotic trees, AFSs create a structurally complex canopy, favorable microclimatic buffering, and an abundant supply of organic matter. Consequently, AFSs act as functional buffer zones between continuous forest patches, such as the adjacent Parque Ecológico Mico-Leão-Dourado and the nearby Reserva Ecológica Poço das Antas (Müller et al. 2022; Silva et al. 2025), and heavily disturbed agricultural or rural matrices, reconciling rural production with ecological restoration (Shennan-Farpón et al. 2022).

Natural phytotelmata accounted for 78.6% of all immatures, reflecting the high availability and long-term ecological stability of these structures, which support a specialized fauna shaped by evolutionary associations (Chathuranga et al. 2023). The higher species richness of natural habitats relative to artificial containers may also reflect their more stable microclimatic conditions, greater availability of organic resources, and complex biotic interactions, including competition and predation, which together increase habitat heterogeneity and niche availability. Rarefaction and extrapolation curves did not reach an asymptote, most likely owing to the high heterogeneity of phytotelm habitats and the absence of sampling from canopy-dwelling bromeliads (Cardoso et al. 2015). Nevertheless, niche partitioning was clearly evident across habitat types.

Bromeliads accounted for the highest species richness (22 species) and overall abundance. Two specialist species, *Cx. pleuristriatus* and *Wy. edwardsi*, together made up 81.7% of the immature mosquitoes found in bromeliads, and both are characteristic of Neotropical Atlantic Forest phytotelmata (Müller and Marcondes 2006). Although both are associated with bromeliad phytotelmata in the Atlantic Forest, their geographic distributions differ: *Cx. pleuristriatus* has a broad Neotropical distribution extending beyond Brazil, with records from several South American countries (Rossi and Lestani 2014), whereas *Wy. edwardsi* has been recorded in Atlantic Forest areas across different Brazilian states, including Rio de Janeiro, Santa Catarina, and Rio Grande do Sul (Marcondes et al. 2003; Alencar et al. 2016).

Bamboo internodes were strongly dominated by *Cx. iridescens* (72.4%), corroborating findings from forest reserves such as Parque Estadual da Cantareira (Ceretti-Junior et al. 2025). Bamboo internodes also supported *Cx. worontzowi*, *Cx. retrosus*, and *Cx. mollis*, highlighting the use of these natural phytotelmata by species with different larval habitat associations. Predatory larvae of *Tx. trichopygus* were also present in these natural microhabitats, where they occupy high trophic levels and may contribute to regulating populations of other mosquito larvae; because the adults are non-hematophagous, *Toxorhynchites* has no direct epidemiological role (Forattini 2002). In contrast, fallen peach palm leaf bracts represented ephemeral phytotelmata with lower richness (9 species), colonized predominantly by *Li. durhamii* and *Ae. albopictus*, with *Ad. squamipennis* also recorded.

Artificial containers harbored reduced species richness (12 species), dominated by synanthropic generalists. Water barrels yielded only 22 individuals representing six species, with *Ae. albopictus* accounting for 63.6% of individuals. Of particular ecological interest were the water-filled plastic tarps, which harbored 1,117 specimens representing nine species, with *Ae. albopictus* and *Li. durhamii* together accounting for 83.7% of individuals. Water-accumulating agricultural plastics are widely used for crop protection and ground cover in rural settings globally, and our findings indicate that plastic tarps act as persistent artificial ground-pool analogues within agroecosystems. We also recorded *Cx. quinquefasciatus* (70 specimens) breeding consistently in plastic tarps within an AFS embedded in preserved forest, a species typically restricted to urban areas or severely degraded urban green spaces (Forattini 2002; Ceretti-Júnior et al. 2014), which indicates increased ecological plasticity under agroforestry management.

The Jaccard dissimilarity analysis reflected these environmental filters: communities in bromeliads and bamboo internodes were the most similar to each other, as were those in peach palm bracts and plastic tarps, whereas water barrels harbored the most distinct community. Oviposition site selection is non-random and is influenced by water chemistry, microclimatic shading, container geometry, and chemical cues (Forattini 2002; Day 2016; Chathuranga et al. 2023).

AFSs are eco-epidemiological interfaces where sylvatic pathogen cycles, vector species, domestic animals, and agricultural workers converge (Rosenstock et al. 2019; Hendy et al. 2023, 2025). Our findings highlight three major epidemiological risks associated with mosquito communities in these systems. First, the colonization of bamboo stands by *Hg. janthinomys* and *Hg. leucocelaenus*, the primary vectors of sylvatic yellow fever virus in the Americas (Abreu et al. 2019), together with *Sa. identicus*, a rarely recorded species considered a secondary yellow fever vector (Lima Bersot et al. 2023), indicates that AFSs containing bamboo can provide larval habitats for sylvatic vector species, potentially increasing opportunities for contact between these mosquitoes and people working in these environments. Second, *Ae. albopictus* was the only species detected across all five larval habitat types sampled, both natural phytotelmata and artificial containers, and its opportunistic behavior and ecological plasticity enable it to exploit both natural forest phytotelmata and anthropogenic containers. This occurrence is epidemiologically relevant, given its recognized potential to act as a bridge vector between sylvatic and anthropogenic transmission cycles (Damasceno-Caldeira et al. 2023). Third, the established presence of *Cx. quinquefasciatus* in agricultural plastic tarps demonstrates how human practices within AFSs can create suitable larval habitats for a synanthropic mosquito species of recognized epidemiological importance.

AFSs are essential tools for biodiversity conservation and sustainable agriculture. However, they can either mitigate or exacerbate vector-borne disease risks depending on their ecological characteristics and management practices (Rosenstock et al. 2019). To minimize public health risks without compromising ecosystem restoration, practical vector management strategies must be integrated into farm maintenance. Farmers should routinely inspect, roll up, or properly dispose of unused plastic tarps and agricultural sheets after rainfall to eliminate artificial larval habitats for *Ae. albopictus* and *Cx. quinquefasciatus*. Water storage barrels must be tightly sealed or fitted with fine mesh screens to prevent female mosquito entry and oviposition. Natural phytotelmata such as bromeliads and bamboo internodes should not be destroyed, as they sustain natural predators such as *Toxorhynchites* larvae and highly specialized, non-vector species that contribute to ecosystem stability and to the competitive displacement of vector taxa.

## Conclusion

This study showed that the Terra Boa Farm AFS, in the Atlantic Forest biome, harbors a diverse immature mosquito fauna, comprising 38 species in 10 genera and including both sylvatic taxa (*Sabethes*, *Wyeomyia*, and *Cx.* (*Microculex*) and species adapted to transitional environments (*Ae. albopictus*, *Limatus*). Species richness exceeded that reported for preserved forest fragments in the region, underscoring the value of AFSs for conserving local mosquito diversity.

Natural larval habitats, particularly bromeliads and bamboo internodes, were the primary drivers of local diversity and harbored most of the species collected. Epidemiologically relevant species occurred across all larval habitat categories, particularly *Ae. albopictus* in both natural and artificial habitats, *Cx. quinquefasciatus* in artificial containers, and *Hg. janthinomys* in natural phytotelmata, which highlights the role of AFSs as interface zones between forest and anthropogenic environments, a characteristic with considerable epidemiological implications given the potential for contact between vector species, human populations, and wildlife reservoirs. Although the species richness recorded here demonstrates the ecological value of AFSs, future studies should incorporate molecular screening for arboviruses and longitudinal assessments of vector-host contact. These approaches would move beyond theoretical assessments of vector potential and allow direct evaluation of arboviral spillover and pathogen transmission at the forest-agriculture interface. Taken together, our findings position AFSs as ecologically complex systems that warrant further investigation to better balance biodiversity conservation and public health surveillance. 

## Supplementary Information

Below is the link to the electronic supplementary material.

Supplementary Figure 1 (PNG 9.90 MB)

High Resolution Image (TIF 2.20 MB)

Supplementary Material 2 (DOCX 14.9 KB)

## Data Availability

I have used data from the online version, which includes supplementary material available at https://doi.org/10.17605/OSF.IO/C2FRA.

## References

[CR1] Abella-Medrano CA, Ibáñez-Bernal S, MacGregor-Fors I, Santiago-Alarcon D (2015) Spatiotemporal variation of mosquito diversity (Diptera: Culicidae) at places with different land-use types within a neotropical montane cloud forest matrix. Parasites vectors 8:487. 10.1186/s13071-015-1086-9 PMC4581103 26399854

[CR2] Abreu FVS, d, Ribeiro IP, Ferreira-de-Brito A, Santos AA, C d, Miranda RM, d, Bonelly IdS, Neves MSAS, Bersot MI, Santos TP, d, Gomes MQ (2019) *Haemagogus leucocelaenus* and *Haemagogus janthinomy*s are the primary vectors in the major yellow fever outbreak in Brazil, 2016–2018. Emerg microbes infections 8:218–231. 10.1080/22221751.2019.1568180 PMC6455131 30866775

[CR3] Alencar J, Mello CF, Serra-Freire NM, Guimarães AÉ, Gil-Santana HR, Gleiser RM (2016) Biodiversity and temporal distribution of immature Culicidae in the Atlantic Forest, Rio de Janeiro State, Brazil. PLoS ONE 11:e0159240. 10.1371/journal.pone.0159240 PMC4942056 27404496

[CR41] Altieri MA, Funes-Monzote FR, Petersen P (2012) Agroecologically efficient agricultural systems for smallholder farmers: contributions to food sovereignty. Agron Sustain Dev 32:1–13. 10.1007/s13593-011-0065-6

[CR4] Barbosa RMR, Sousa EMDE, Faierstein GB, Luna CF, Oliveira, A L S D E, Paiva MHS (2025) Diversity and Spatial Distribution of Mosquitoes (Culicidae) in Forest-Urban Transition Areas of an Atlantic Forest Fragment in Pernambuco, Brazil. An Acad Bras Cienc 97:e20250139. 10.1590/0001-3765202520250139 41172404

[CR5] Calderón-Peláez M-A, Mantilla-Granados JS, Velandia-Romero ML, Calvo E, Castellanos JE, Olano VA (2023) A strategy for entomo-virological surveillance of yellow fever, dengue, zika, and chikungunya viruses in field-collected mosquitoes. MethodsX 11:102356. 10.1016/j.mex.2023.102356 PMC10493583 37701736

[CR6] Camara DCP, da Silva Pinel C, Rocha GP, Codeco CT, Honorio NA (2020) Diversity of mosquito (Diptera: Culicidae) vectors in a heterogeneous landscape endemic for arboviruses. Acta Trop 212:105715. 10.1016/j.actatropica.2020.105715 32971068

[CR7] Cardoso CAA, Lourenço-de-Oliveira R, Codeço CT, Motta MA (2015) Mosquitoes in bromeliads at ground level of the Brazilian Atlantic Forest: the relationship between mosquito fauna, water volume, and plant type. Ann Entomol Soc Am 108:449–458. 10.1093/aesa/sav040 PMC4765313 27418695

[CR8] Catenacci L, Nunes-neto J, Castro FC, Lemos P, Oyama E, Deem SL, Travassos-da-Rosa E (2017) New records of mosquito species (Diptera: Culicidae) for Bahia (Brazil). Int J Mosq Res 4:12–16

[CR9] Catenacci LS, Nunes-Neto J, Deem SL, Palmer JL, Travassos‐da Rosa ES, Tello JS (2018) Diversity patterns of hematophagous insects in Atlantic forest fragments and human‐modified areas of southern Bahia, Brazil. J Vector Ecol 43:293–304. 10.1111/jvec.12313 30408294

[CR11] Ceretti-Júnior W, Medeiros-Sousa AR, Multini LC, Urbinatti PR, Vendrami DP, Natal D, Marques S, Fernandes A, Ogata H, Marrelli MT (2014) Immature mosquitoes in bamboo internodes in municipal parks, city of São Paulo, Brazil. J Am Mosq Control Assoc 30:268–274. 10.2987/14-6403R.1 25843132

[CR10] Ceretti-Junior W, Medeiros-Sousa AR, de Paula MB, Evangelista E, Barrio-Nuevo KM, Wilk-da-Silva R, Oliveira-Christe R, Marrelli MT (2025) Species Composition and Ecological Aspects of Immature Mosquitoes (Diptera: Culicidae) in Phytotelmata in Cantareira State Park, São Paulo, Brazil. Insects 16:376. 10.3390/insects16040376 PMC12027585 40332835

[CR12] Chathuranga WGD, Weeraratne TC, Abeysundara SP, Karunaratne P, de Silva, W A P P (2023) Breeding site selection and co-existing patterns of tropical mosquitoes. Med Vet Entomol 37:550–561. 10.1111/mve.12656 37060294

[CR13] Consoli RAGB, Oliveira RLd (1994) Principais mosquitos de importância sanitária no Brasil. Editora da Fundação Oswaldo Cruz, Rio de janeiro, p 228

[CR14] Damasceno-Caldeira R, Nunes-Neto JP, Aragão CF, Freitas MNO, Ferreira MS, Castro PHG, d, Dias DD, Araújo PA, d S, Brandão RCF, Nunes BTD, Silva, E V P d, Martins LC, Vasconcelos (2023) Vector competence of *Aedes albopictus* for yellow fever virus: risk of reemergence of urban yellow fever in Brazil. Viruses 15:1019 P F d C, Cruz, A C R. 10.3390/v15041019 PMC10146658 37112999

[CR15] Daudt-Lemos M, Ramos-Silva A, Faustino R, Noronha TG, d, Vianna RA, d O, Cabral-Castro MJ, Cardoso CAA, Silva AA, Carvalho FR (2025) Rising incidence and spatiotemporal dynamics of emerging and reemerging arboviruses in Brazil. Viruses 17:158. 10.3390/v17020158 PMC11860164 40006913

[CR16] Day JF (2016) Mosquito oviposition behavior and vector control. Insects 7:65. 10.3390/insects7040065 PMC5198213 27869724

[CR17] Forattini OP (2002) Culicidologia Médica: Identificação, Biologia, Epidemiologia. EDUSP, São Paulo, p 860p

[CR18] Gerberg EJ, Barnard DR, Ward RA (1994) Manual for mosquito rearing and experimental techniques. American Mosquito Control Association Bulletin, 5. Lake Charles, Louisiana, American Mosquito Control Association. 98 p

[CR40] Gliessman SR (2000) Agroecologia: processos ecológicos em agricultura sustentável. Universidade/UFRGS, Porto Alegre, 653 p

[CR20] Hendy A, Hernandez-Acosta E, Valério D, Fé NF, Mendonça CR, Costa ER, Andrade ES, d A, Júnior JT, Assunção FP, Scarpassa VM, Guimarães de Lacerda MV, Buenemann M, Vasilakis N, Hanley KA (2023) Where boundaries become bridges: Mosquito community composition, key vectors, and environmental associations at forest edges in the central Brazilian Amazon. PLoS Negl Trop Dis 17:e0011296. 10.1371/journal.pntd.0011296 PMC10166490 37099599

[CR19] Hendy A, Fé NF, Pedrosa I, Girão A, dos Santos TN, F, Mendonça CR, Andes Júnior JT, Assunção FP, Costa ER, Sluydts V, Gordo M, Scarpassa VM, Buenemann M, de Guimarães MV, Gomes Mourão MP, Vasilakis N, Hanley KA (2025) Forest edge landscape context affects mosquito community composition and risk of pathogen emergence. Iscience 28:111576. 10.1016/j.isci.2024.111576 PMC11758831 39868037

[CR21] Hsieh TC, Ma KH, Chao A (2016) iNEXT: an R package for rarefaction and extrapolation of species diversity (Hill numbers). Methods Ecol Evol 7:1451–1456. 10.1111/2041-210X.12613

[CR22] Lane J (1953) Neotropical Culicidae. São Paulo, University of São Paulo, p 1112

[CR23] Lima Bersot MI, Vieira G, De Moraes JR, Rocha Pereira G, Albuquerque Motta M, Lourenço-De-Oliveira R (2023) Biological and behavioral features and colonization of the sylvatic mosquito *Sabethes identicus* (Diptera: Culicidae). PLoS ONE 18:e0296289. 10.1371/journal.pone.0296289 PMC10735041 38128039

[CR24] Marchi MJ, Müller GA, Marcondes CB (2010) Mosquitos (Diptera: Culicidae) de uma futura Unidade de Conservação em área de Mata Atlântica no sul do Brasil. EntomoBrasilis 3:34–37. 10.12741/ebrasilis.v3i2.77

[CR25] Marcondes CB, Fernandes A, Paterno U, Müller GA, de Pinho LC, Struffaldi DV (2003) New records of mosquitoes from the southern Brazilian States of Santa Catarina and Rio Grande do Sul, with 18 species new for the States (Diptera: Culicidae). Zootaxa 347:1–6. 10.11646/zootaxa.347.1.1

[CR26] Martínez-Barciela Y, Polina A, Garrido J (2025) Habitat characterization and breeding preferences of mosquito larvae in northwestern Spain: abundance, diversity, and species composition. Parasites Vectors 18:165. 10.1186/s13071-025-06803-1 PMC12057168 40329353

[CR29] Müller GA, Marcondes CB (2006) Bromeliad-associated mosquitoes from Atlantic forest in Santa Catarina Island, southern Brazil (Diptera, Culicidae), with new records for the State of Santa Catarina. Iheringia Série Zoologia 96:315–319. 10.1590/S0073-47212006000300007

[CR28] Müller GA, Marchi MJ, Marcondes CB (2014) Mosquito immatures in bamboo internodes in eastern Santa Catarina State, South Brazil (Diptera Culicidae). Biotemas 27:151–154. 10.5007/2175-7925.2014v27n1p151

[CR27] Müller GA, de Mello CF, Bueno AS, de Alcantara Azevedo WT, Alencar J (2022) Little noticed, but very important: The role of breeding sites formed by bamboos in maintaining the diversity of mosquitoes (Diptera: Culicidae) in the Atlantic Forest biome. PLoS ONE 17:e0273774. 10.1371/journal.pone.0273774 PMC9447929 36067179

[CR30] Oliveira-Christe R, Marrelli MT (2025) Descriptions of the male genitalia of species of *Culex* (*Microculex*) (Diptera: Culicidae) from the Atlantic Forest, Brazil, and a dichotomous identification key for those species. Zootaxa 5717:517–543. 10.11646/zootaxa.5717.4.3 42407494

[CR31] R Core Team (2025) R: A language and environment for statistical computing. R Foundation for Statistical Computing, Vienna, Austria

[CR32] Reinert JF (2009) List of abbreviations for currently valid generic-level taxa in family Culicidae (Diptera). Eur Mosq Bull 27:68–76

[CR33] Rosenstock TS, Dawson IK, Aynekulu E, Chomba S, Degrande A, Fornace K, Jamnadass R, Kimaro A, Kindt R, Lamanna C, Malesu M, Mausch K, McMullin S, Murage P, Namoi N, Njenga M, Nyoka I, Paez Valencia AM, Sola P, Shepherd K, Steward P (2019) A Planetary Health Perspective on Agroforestry in Sub-Saharan Africa. One Earth 1:330–344. 10.1016/j.oneear.2019.10.017

[CR34] Rossi GC, Lestani EA (2014) New records of mosquitoes (Diptera: Culicidae) from Misiones Province, Argentina. Revista de la Sociedad Entomológica Argentina 73:49–53

[CR35] Shennan-Farpón Y, Mills M, Souza A, Homewood K (2022) The role of agroforestry in restoring Brazil’s Atlantic Forest: Opportunities and challenges for smallholder farmers. People Nat 4:462–480. 10.1002/pan3.10297

[CR38] Silva SOF, Mello CF, Pavão LdM, Gil-Santana HR, Alencar J (2024) Ecological Importance of Breeding Sites in Atlantic Forest Fragments: A Focus on Culicidae Diversity with Particular Attention to Vector Species. Rev Soc Bras Med Trop 57:e007152024. 10.1590/0037-8682-0108-2024 PMC11654466 39699543

[CR37] Silva SOF, Mello CF, Maia D, Alencar J (2025) Vertical and Temporal Distribution of Medically Important Mosquito Species In an Atlantic Forest Fragment of Southeastern Brazil: Ecological Insights and Public Health Implications. J Am Mosq Control Assoc 41:61–68. 10.2987/25-7217 40683627

[CR36] Silva JdS, Mello CF, Freitas Silva SO, Bastos AQ, Carbajal-de-la-Fuente AL, Araújo TR, Alencar J (2026) Oviposition Activity and Ecological Patterns of Medically Important Culicidae in an Agroforestry System in the Atlantic Forest, Rio de Janeiro, Brazil. Trop Med Infect Disease 11:210. 10.3390/tropicalmed11080210 PMC13517476 42646787

[CR39] Zha X, Zhang Z (2025) Opinionated views on biophysical and social constraints on agroforestry system. Front Plant Sci 16:1607207. 10.3389/fpls.2025.1607207 PMC12390968 40894510

