# Supplementary material for: Phytotelm and anthropogenic mosquitoes: community structure in natural and artificial habitats in an Atlantic Forest agroforestry system, Rio de Janeiro, Brazil
Source: Naturwissenschaften. 2026 Sep 16;113(5):113. doi: 10.1007/s00114-026-02162-8 (PMC13582279; doi:10.1007/s00114-026-02162-8)
Supplement: Supplementary file 1 [file 114_2026_2162_MOESM2_ESM.docx]

Table S1. Complementary matrix of mosquito species occurrences by breeding habitat. List of mosquito species associated with each natural and artificial breeding container, complementing the network analysis presented in Figure 4.

|  | Bromeliad | Bamboo | Palm | Plastic canvas | Water tank |
| --- | --- | --- | --- | --- | --- |
| Bromeliad | **22 (10)** | 12 | 6 | 5 | 4 |
| Bamboo |  | **21 (8)** | 6 | 5 | 3 |
| Palm |  |  | **9 (2)** | 5 | 2 |
| Plastic canvas |  |  |  | **9 (2)** | 3 |
| Water tank |  |  |  |  | **6 (1)** |
